# The key role of microRNA-766 in the cancer development

**DOI:** 10.3389/fonc.2023.1173827

**Published:** 2023-05-02

**Authors:** Jitendra Gupta, Hussein Riyadh Abdul Kareem Al-Hetty, Murtadha Sh. Aswood, Abduladheem Turki Jalil, Maha Dhurgham Azeez, Zafar Aminov, Fahad Alsaikhan, Andrés Alexis Ramírez-Coronel, Pushpamala Ramaiah, Bagher Farhood

**Affiliations:** ^1^ Institute of Pharmaceutical Research, GLA University, Mathura, India; ^2^ Department of Nursing, Al-Maarif University College, Ramadi, Anbar, Iraq; ^3^ Department of Physics, College of Education, University of Al-Qadisiyah, Al-Diwaniyah, Iraq; ^4^ Medical Laboratories Techniques Department, Al-Mustaqbal University College, Babylon, Hilla, Iraq; ^5^ National University of Science and Technology, Dhi Qar, Iraq; ^6^ Department of Public Health and Healthcare management, Samarkand State Medical University, Samarkand, Uzbekistan; ^7^ Department of Scientific Affairs, Tashkent State Dental Institute, Tashkent, Uzbekistan; ^8^ College of Pharmacy, Prince Sattam Bin Abdulaziz University, Alkharj, Saudi Arabia; ^9^ Azogues Campus Nursing Career, Health and Behavior Research Group (HBR), Psychometry and Ethology Laboratory, Catholic University of Cuenca, Cuenca, Ecuador; ^10^ Epidemiology and Biostatistics Research Group, CES University, Medellín, Colombia; ^11^ Educational Statistics Research Group (GIEE), National University of Education, Azogues, Ecuador; ^12^ Faculty of Nursing, Umm al-Qura University, Makkah, Saudi Arabia; ^13^ Department of Medical Physics and Radiology, Faculty of Paramedical Sciences, Kashan University of Medical Sciences, Kashan, Iran

**Keywords:** cancer, microRNA, miR-766, biomarker, drug resistance

## Abstract

Cancer is caused by defects in coding and non-coding RNAs. In addition, duplicated biological pathways diminish the efficacy of mono target cancer drugs. MicroRNAs (miRNAs) are short, endogenous, non-coding RNAs that regulate many target genes and play a crucial role in physiological processes such as cell division, differentiation, cell cycle, proliferation, and apoptosis, which are frequently disrupted in diseases such as cancer. MiR-766, one of the most adaptable and highly conserved microRNAs, is notably overexpressed in several diseases, including malignant tumors. Variations in miR-766 expression are linked to various pathological and physiological processes. Additionally, miR-766 promotes therapeutic resistance pathways in various types of tumors. Here, we present and discuss evidence implicating miR-766 in the development of cancer and treatment resistance. In addition, we discuss the potential applications of miR-766 as a therapeutic cancer target, diagnostic biomarker, and prognostic indicator. This may shed light on the development of novel therapeutic strategies for cancer therapy.

## 1 Introduction

The significance of post-transcriptional gene regulation in the development and spread of cancer has recently become much clearer. Although messenger RNA (mRNA) levels in cells are significantly influenced by transcriptional activation and repression, posttranscriptional processes ultimately decide whether or not mRNA will be translated into proteins. These would include microRNAs’ negative regulation and RNA-binding proteins’ control of RNA longevity (miRNAs) (1–8). Noncoding RNAs known as miRNAs are a conserved family that is named for their short size (18–22 nucleotides on average) (9–17). According to experts, miRNAs can control most (minimum of 2/3) of protein-coding genes (18). circular RNAs and Long non-coding RNAs are two examples of non-protein-coding transcripts that may be associated with miRNAs (19). They are found in almost all tissues (18). The RNA-induced silencing complex (RISC) is produced when specialised protein factors, including Argonaute proteins, bind to microRNAs to exert a negative regulation on certain target genes. The RISC detects target transcripts by a direct link between miRNA and mRNA molecules, often based on patterns in the 3’ untranslated regions (3’ UTR) of the mRNA. By encouraging mRNA instability, preventing translation, or directly cleaving mRNA, expression is suppressed (20). RNA polymerase II frequently transcripts miRNAs from distinct genomic components (21). But introns that are in protein-coding genes contain hairpin structures that can also produce miRNAs (22, 23).

Every single miRNA may simultaneously target dozens or even hundreds of genes seed sequence refers to the first eight nucleotides of the miRNA, which are essential for target identification (20). MiRNAs regulate gene expression, leading to dysregulated expression and abnormal activity (24–29). MiRNAs regulate a variety of biological processes, such as cell development, differentiation, proliferation, apoptosis, tumorigenesis, cancer progression, and signal transduction, based on the targeted genes, such as tumour inhibitor genes and oncogenes (30–35). Additionally, miRNAs could be therapeutic in addition to diagnostic biomarkers (36, 37). Ever since the finding of circulating miRNAs in 2008, >79 miRNAs have been identified as serum or plasma biomarkers of a variety of malignancies, such as gastric, lung, breast, esophageal, colon, melanoma, ovarian, and prostate cancer (38–45). One important miRNA, miR-766, is found in an intron that exists in the Septin-6 (SEP6) gene and promotes the course of cancer (46). MiR-766 expression is linked to carcinogenesis, malignant behavior, and tumor suppressive and/or oncogene genes (47–53). In addition to hematological malignancies, miR-766 is implicated in several types of cancers, such as colon, colorectal, papillary thyroid, prostate, cervical, breast, osteosarcoma, lung, and gastric cancer. MiR-766 is an oncogene or tumour-inhibiting gene, and its function in the genesis of malignancies is discussed.

## 2 MicroRNA biogenesis

RNA polymerase II transcribes microRNAs to create a double-stranded hairpin primary transcript. Pre-miRNA is broken down by Drosha and converted into mature miRNA duplex strands by RNase III endonuclease Dicer (54–57). Strands are oriented in either 5’ or 3’ orientation. Argonaute can be loaded with either RNA strand to form RISC, with the ratio of -5p to -3p dependent on thermodynamic endurance (58). But, depending on the kind of cell, the ratio of -5p to -3p may favor one strand to the other or be equal. RISC can be tailored to different gene targets depending on the number or presence of different strands (59, 60). When miRNAs attach to their mRNA targets’ 3’-UTRs by complementary base pairing, they subsequently regulate gene expression by either targeting their destruction or repressing translation. Up to sixty percent of the human genome’s genes are controlled by microRNAs, which have been discovered to impact a number of cellular processes and the onset of illness (61–68). Additionally, miRNAs and pre-miRNAs are highly firm in the extracellular environment and can be produced from the cell through into bloodstream. Because they are carried inside exosomes, circulating microvesicles, protein complexes, or high-density lipoproteins, they may be taken up by cells in tissues through cell-to-cell communication (69). They are becoming clinical biomarkers and therapeutic targets for individualised medicine in sophisticated diseases, and they continue to stimulate a wide range of research (24, 25, 29, 70, 71).

## 3 miRNA acts as OncomiR or tumor suppressor

MicroRNAs can inhibit or promote the cancer phenotype by inhibiting the production of oncogenes or tumour suppressors (25, 72–77). Typically, tumor-suppressive miRNAs are expressed less than it should while oncogenic miRNAs (oncomiRs) are expressed more than enough in malignancies (71, 78–85). Based on the kind of cancer and the particular miRNA that is impacted, cancer progression, metastasis, and/or survival could be drastically decreased whenever these oncomiRs or miRNAs suppress tumors are silenced or activated, respectively. It is also conceivable for malignancies to become entirely dependent on, or “addicted” to, an oncomiR, in which case suppressing the oncomiR causes the tumour to fully regress (86). Therefore, miRNAs may be classed as either tumor-promoting or tumor-suppressing, and the regulation of their synthesis for therapeutic purposes is a fiercely disputed subject (87–91).

**Figure 1:**
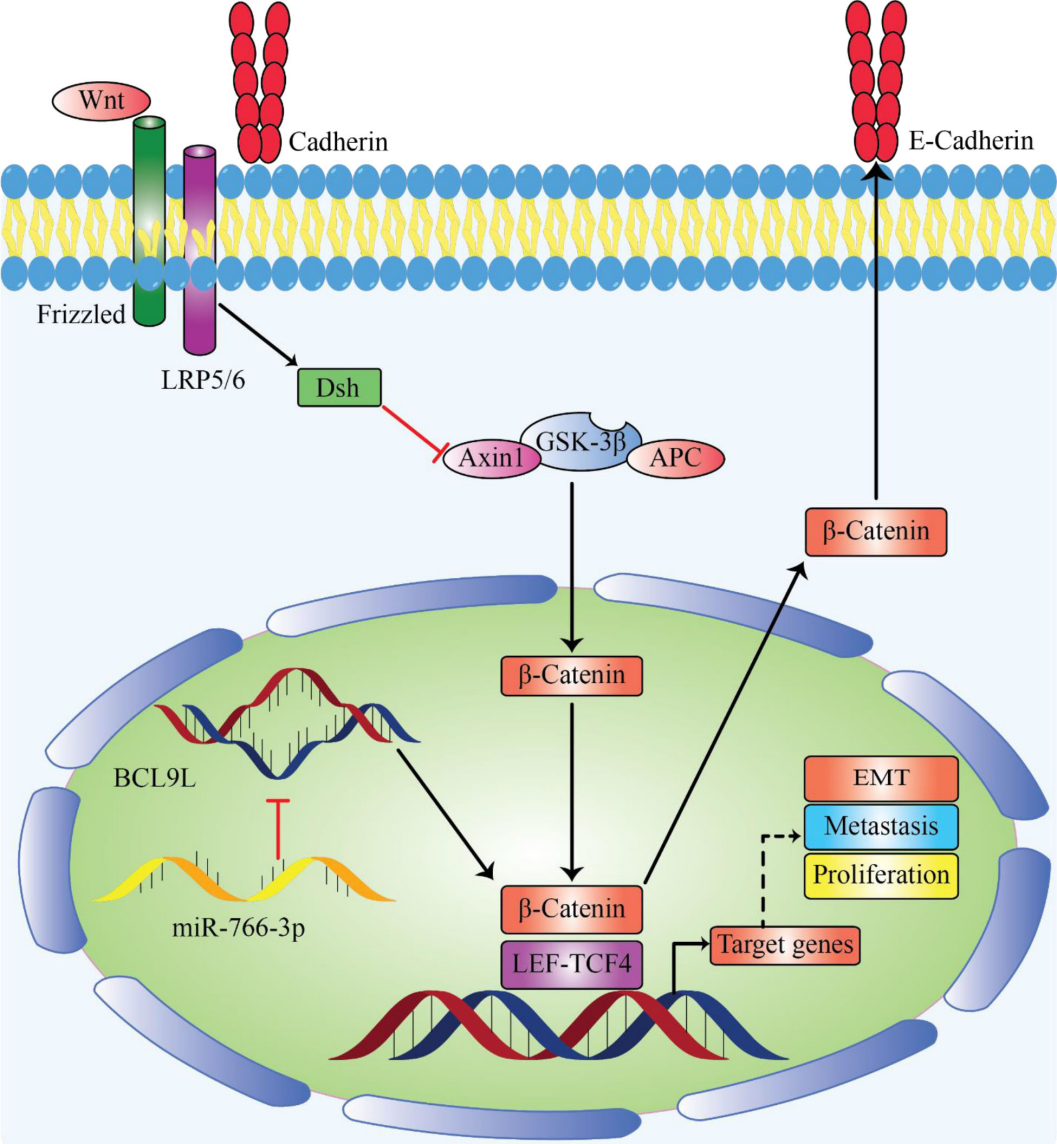
MiR-766 can be sererved as tumor suppressor miRNA by decreasing cancer development. Enforced expression of miR-766-3p lead to suppression of metastasis by inhibiting EMT, migration and invasion in osteosarcoma by regulating the β-Catenin/TCF-4 signal pathway and targeting BCL9L (92).

Yet, there are arguments in favor of caution in the therapeutic approaches. There are a lot of contradictory reports in the literature about whether or not particular miRNAs are tumor-suppressing or carcinogenic. MiRNAs can be both tumor suppressive and carcinogenic. For example, miR-766 can act as both a tumor suppressor (**Figure 1**) and oncomiR in osteosarcoma (51, 92). Given the vast array of genes that a certain miRNA affects, the variety of consequences is not surprising. It implies that any designation of a miRNA as oncogenic or tumor suppressive must be carefully examined because it may constitute an oversimplification (93).

## 4 miR-766

### 4.1 miR-766 as tumor suppressor miRNA in cancer cells

#### 4.1.1 Colorectal cancer

Colorectal cancer (CRC) is the third most prevalent malignancy and the third leading cause of cancer-related death worldwide (94, 95). CRC affects 4.4% of persons in affluent nations and 1.4% of people in underdeveloped nations (96). Despite improvements in care over the past few decades, almost 50% of CRC patients pass away within 5 years. Surgical excision causes a high mortality rate due to tumour reappearance and metastasis (97, 98). Therefore, continued research further into underlying molecular pathways of CRC is very important from a therapeutic standpoint.

A group of transcription factors known as nuclear receptors (NRs) controls homeostasis, metabolism and differentiation by spatiotemporally regulating gene expression. Humans only have 48 NRs that have been found, 25 of them are orphan NRs that have unknown endogenous biological roles and ligands (99, 100). Numerous studies have demonstrated the tight connection between orphan NRs and specific forms of cancer (101). The orphan nuclear receptor hepatocyte nuclear factor 4 gamma (HNF4G) is substantially elevated in CRC tissues (102). HNF4 function is still not entirely understood, yet. Although, dysregulation of miR-766-3p level in CRC has been documented (**Table 1**), which suggests it may be crucial in CRC pathogenesis.

**Table 1:** Effect of miR-766 on the cancer cells.

Cancer type	Expression (Oncomir/tumor suppressor)	Target of miR-766	Sample (Human, *In vitro*, *In vivo*)	Note	Ref
CRC	Up (Oncomir)	TRIM67	Human (CRC tissue), *In vitro* (HCT116 cells)	- TRIM67 may act as an anticancer factor during CRC. - miR-766-5p acts as an oncomiR in CRC by targeting TRIM67. - Exosomal circ_0094343 promotes the chemosensitivity of CRC cells and suppresses the glycolysis and proliferation of CRC cells by sponging miR-766-5p.	(76)
CRC	Up (Oncomir)	SOX6	Human (CRC tissue), *In vitro* (SW480 cells)	- miR-766 acts as oncomiR and contribute to CRC development by targeting SOX6.	(47)
CRC	Up (Oncomir)	SCAI	Human (CRC tissue), *In vitro* (SW480 cells)	- Inhibition of miR−766−5p decreases invasion, migration, and proliferation of CRC, as well as promotes apoptosis of cancer cells.	(103)
Breast cancer	- (Oncomir)	PTEN	*In vitro* (MCF-7 and T47D cells), *In vivo* (tumor xenograft model)	- Overexpressed miR-766 induces migration, invasion, and proliferation of breast cancer cells as well as lead to decreases sensitivity of breast cancer cells to treatment with 5-fluorouracil. - miR-766 promotes cancer growth *in vivo*.	(48)
CSCC	Up (Oncomir)	HOXA9	Human (CSCC tissues), *In vitro* (CSCC cell line)	- Upregulated circFADS2 suppresses proliferation, glycolysis and metastasis of CSCC cell by regulation of HOXA9 through sponging miR-766-3p.	(49)
CSCC	Up (Oncomir)	PDCD5	Human (CSCC tissues), *In vitro* (A431, SCL-1 and DJM-1 cells), *In vivo* (mice)	- miR-766 can inhibit apoptosis and induce invasion, migration, and proliferation of SCL-1 and A431 cells by decreasing the expression level of PDCD5. - Inhibition of miR-766 decreases tumor growth in mice.	(104)
Chronic myeloid leukemia	Up (Oncomir)	CDKN1A	Human (Blood), *In vitro* (KCL22 and K562 cells)	- lncRNA-NEAT1 inhibits proliferation and promotes apoptosis of CML Cells by sponging miR-766-5p. In return, upregulated miR-766-5p reversing the NEAT1 effects on apoptosis and Viability of the CML Cells	(105)
APL	- (Oncomir)	Caspase-3 and Bax	*In vitro* (HL-60 and NB4)	- Overexpressed miR-766 can contribute to APL development through increasing cell viability by targeting caspase-3 and Bax.	(106)
HCC	Up (Oncomir)	NR3C2	Human (HCC cancer tissue), *In vitro* (SMMC-7721), *In vivo* (BALB/c nude mice)	- miR-766 act as oncomiR in HCC through including metastasis and proliferation of HCC cells by targeting NR3C2.	(50)
Osteosarcoma	Up (Oncomir)	YTHDF2	Human (Osteosarcoma tissue), *In vitro* (MG63 and U2OS), *In vivo* (tumor xenograft model)	- circ_0001105 can inhibit the development of osteosarcoma by sponging miR-766. - Overexpressed miR-766 promotes colony formation ability and viability of osteosarcoma cells by targeting YTHDF2.	(51)
Cervical cancer	Up (Oncomir)	SCAI	Human (Cervical cancer tissue and serum), *In vitro* (SiHa cell), *In vivo* (mice injected with SiHa cell)	- Inhibition of miR-766-5p lead to induction of apoptosis and suppression of proliferation, invasion and migration of cervical cancer cells by downregulation SCAI level.	(107)
Gastric cancer	Up (–)	–	Human (gastric cancer obtained from patients with stage IV GCa who received platinum-based chemotherapy),	- The expression level of miR-766 was significantly upregulated in patients with gastric cancer who have better chemotherapy responses and more prolonged progression-free survival.	(108)
Colon cancer	Down (tumor suppressor)	TGFBI	*In vitro* (Caco-2, SW480, HCT116 and SW620)	- Overexpressed miR-766-3p promotes apoptosis of colon cancer cells and inhibits cancer progression by targeting TGFBI.	(53)
Colon cancer	- (tumor suppressor)	DNMT3B	*In vitro* (HCT116 and SW480)	- Overexpression of miR-766 contribute to inhibition of colon cancer development by decreasing methylation of tumor suppressor genes including DKK2, WIF1, SFRP1, and SFRP2.	(109)
Colon Cancer	Down (tumor suppressor)	–	Human (colon cancer tissue), *In vitro* (Caco2 cells)	- miR-766 as tumor suppressor miRNA in colon cancer cells, promotes apoptosis through regulating the p53/Bax signaling pathway by MDM4.	(110)
CRC	- (tumor suppressor)	HNF4G	Human (CRC tissues), *In vitro* (HCT116, SW620, LOVO, HT29, and SW480)	- miR-766-3p suppresses CRC cell proliferation by targeting HNF4G and inhibits PI3K/AKT pathway.	(111)
Breast cancer	- (tumor suppressor)	MDM4	*In vitro* (SBC3, U2OS cells)	- Overexpressed miR-766 inhibits breast cancer progression by enhancing p53 signaling through targeting MDM4.	(52)
Breast cancer	- (tumor suppressor)	–	*In vivo* (NOD/scid/IL2Rγ ^−/−^ mice)	- Upregulated miR-766 significantly reduces lung metastasis during breast cancer.	(112)
HCC	Down (tumor suppressor)	–	Human (HCC tissues), *In vitro* (SMMC7721, SK-HEP-1, Hep3B, PLC/PRF/5 and Huh7)	- miR-766-3p acts as tumor suppressor miRNA in HCC, maybe by targeting the Wnt3a/PRC1 pathway.	(113)
HCC	- (tumor suppressor)	MTA3	Human (HCC cancer tissue), *In vitro* (HepG2 and Huh-7 cells), *In vivo* (BALB/c nude mice)	- Circ_0021093 inhibits apoptosis as well as induces invasion, migration and cell growth in HCC cells by sponging miR-766-3p and increasing MTA3 expression.	(114)
HCC	Down (tumor suppressor)	FOSL2	Human (HCC tissues), *In vitro* (SNU449 and SK-HEP-1 cells), *In vivo* (tumor xenograft model)	- Silencing miR-766-3p enhances the cell growth, invasive, and migration properties of HCC cells by targeting FOSL2. In return, upregulated circ_0056836 promotes the invasive and migration properties of HCC cells by sponging miR-766-3p.	(115)
RCC	Down (tumor suppressor)	SF2	Human (RCC tissue), *In vitro* (OSRC-2, A498, ACHN, 786-O)	- miR-766-3p promoter is highly methylated in RCC cells. - Overexpression of miR-766-3p lead to inhibition of cell-cycle progression in RCC cells by targeting SF2.	(116)
Primary lung carcinoma	Up (tumor suppressor)	–	Human (primary lung carcinoma tissue), *In vitro* (A549 and SPC-A-1 cells)	- CircZNF652 induces migration and proliferation of primary lung carcinoma cells by sponging miR-766.	(117)
Lung cancer	Down (tumor suppressor)	–	Human (lung cancer tissue), *In vitro* (A549, SPC-A-1, H1975, and H1299 cells), *In vivo* (BALB/c nude mice)	- miR-766-5p act as tumor suppressor miRNA in lung cancer. - Ectopic expression of lncRNA CASC15 contribute to ling cancer progression by targeting miR-766-5p/KLK12 axis.	(118)
LUAD	Down (tumor suppressor)	MAPK1	Human (LUAD tissue), *In vitro* (A549 and SPC-A1 cells), *In vivo* (tumor xenograft model)	- lncRNA-PRKCZ-AS1 induces the LUAD tumorigenesis by sponging miR-766-5p to regulation MAPK1.	(119)
LSCC	Down (tumor suppressor)	HMGA2	*In vitro* (AMC-HN-8 and TU686)	- circSHKBP1 induces LSCC tumorigenesis through overexpressing HMGA2 by sponging miR-766-5p.	(120)
PDAC	Down (tumor suppressor)	ETS1	Human (PDAC tissue), *In vitro* (Aspc−1 and Panc−1 cells)	- Upregulated miRNA−766 lead to inhibition of proliferation and invasion of PDAC cells.	(121)
PCa	Down (tumor suppressor)	E2F3	Human (PCa tissues), *In vitro* (LNCaP, PC3, DU145, P4E6).	- Overexpressed miR-766-5p suppresses invasion, migration and proliferation of PCa cells by targeting E2F transcription factor 3 (E2F3). In return, lncRNA-NEAT1 promotes PCa development by sponging miR-766-5p.	(122)
Pancreatic cancer	Down (tumor suppressor)	ATG7	*In vitro* (PC cells), *In vivo* (mice)	- CircATG7 induces autophagy, motility, proliferation, and metastasis of pancreatic cancer by miR-766-5p/ATG7 and HUR/ATG7 axis.	(123)
Osteosarcoma	Down (tumor suppressor)	BCL9L	Human (Osteosarcoma tissue), *In vitro* (MG-63, HOS, U2OS, Saos-2, and 143B), *In vivo* (tumor xenograft model)	- Upregulated miR-766-3p suppresses migration and invasion of osteosarcoma by targeting BCL9L.	(92)
Bladder cancer	Down (tumor suppressor)	MTA1	Human (Bladder cancer tissue), *In vitro* (T24 and J82)	- LINC00963 promotes ovarian carcinoma progression by upregulating PD-L1. - MiR-766-3p is a tumour suppressor in bladder cancer.	(124)
Cancer cells	Down (tumor suppressor)	CBP and BRD4	*In vitro* (HCT116^+/+^, HCT116^−/−^, MIAPaCa2, AGS, KYSE150, and HSC2 cells), *In vivo* (xenograft mouse model of HCT116^−/−^ cells)	- miR-766-5p decreases the MYC expression level in cancer cells. - Forced expression of miR-766-5p lead to inhibition of tumor growth by targeting CBP and BRD4.	(125)
Ovarian carcinoma resistance to carboplatin	Down (tumor suppressor)	PD-L1	*In vitro* (OVCAR-3/CBP and CAOV-3/CBP cells).	- LINC01503 promotes ovarian carcinoma progression by upregulating PD-L1.	(87)
PTC	Down (tumor suppressor)	–	*In vitro* (PTC cells)	- lncRNA-MPEG1-1 acts as an oncogene and promotes the proliferation and metastasis of PTC cells by sponging miR-766-5p. - Cancer development partially reversed through upregulated miR-766-5p in PTC cells.	(126)
PTC	Down (tumor suppressor)	ARFGEF1	Human (PTC tissue), *In vitro* (PTC cells), *In vivo* (murine xenograft model)	- circ_0059354 induces cancer development through overexpression of ARFGEF1 by targeting miR-766-3p and also circ_0059354 deficient inhibits tumor growth. - miR-766-3p acts as tumor suppressor miRNA by targeting ARFGEF1.	(80)
NSCLC	Down (tumor suppressor)	FAM83A	Human (tissue)	- CircMIIP induces cancer development by increasing the expression level of FAM83A through sponging miR-766-5p.	(127)

CRC, colorectal cancer cells; PTC, Papillary thyroid cancer; circMIIP, Circular RNA migration and invasion inhibitory protein; NSCLC, non-small cell lung cancer; HNF4G, hepatocyte nuclear factor 4 gamma; PCa, Prostate cancer; LSCC, Laryngeal squamous cell carcinoma; CBP, Ovarian carcinoma resistance to carboplatin; CSCC, cutaneous squamous cell carcinoma; HCC, hepatocellular carcinoma; RCC, renal cell carcinoma; APL, acute promyelocytic leukemia; PDAC, pancreatic ductal adenocarcinoma; LUAD, Lung adenocarcinoma; CSCC, cutaneous squamous cell carcinoma.

**Figure 2:**
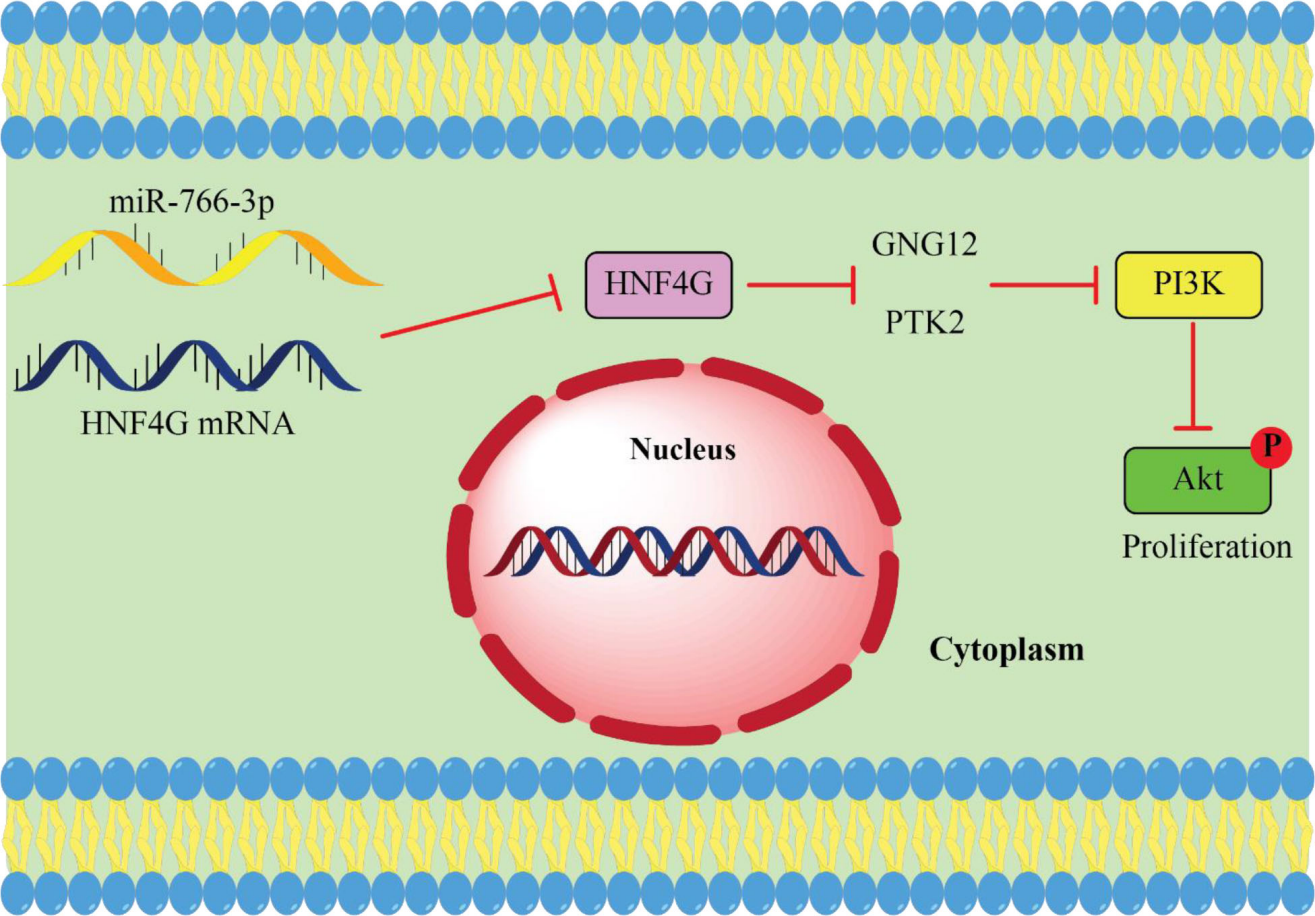
miR-766-3p acts as tumor suppressor miRNA in CRC. upregulated HNF4G leads to cancer cell proliferation by activating the PI3K/AKT pathway in return miR-766 suppresses CRC cell proliferation by targeting HNF4G and inhibits PI3K/AKT pathway (111).

Recently, He et al. (111) HNF4G expression is noticeably elevated in CRC tissues. In addition, there was a definite association between a high degree of tumor-node metastasis and a poor prognosis for CRC, as well as an increased expression of HNF4G. Additionally, HNF4G overexpression strongly induced CRC cell proliferation *in vitro*. In addition, they identified that HNF4G targets G protein gamma 12 (GNG12) and protein tyrosine kinase 2 to activate the phosphatidylinositol 3-kinase/protein kinase B (PI3K/AKT) pathway and enhance cell proliferation in colorectal cancer (CRC) (PTK2). MiR-766-3p expression was significantly downregulated and elevated in CRC. Both of these changes were associated with miR-766-3p ability’s to target HNF4G *in vivo* and *in vitro* and prevent CRC cell proliferation. Collectively, their findings demonstrated that miR-766-3p suppresses the phosphatidylinositol 3−kinase (PI3K)/protein kinase B (AKT) pathway by targeting the expression of HNF4G, which in turn limits the growth of CRC cells (111). As a result, CRC treatment may benefit from the development of medicines that specifically aim at the miR-766-3p/HNF4G axis (**Figure 2**).

#### 4.1.2 Colon cancer

With more than 1,800,000 cases reported and more than 881,000 deaths in 2018, colon cancer ranks third among the most common malignancies globally, accounting for about 10 percent of all cancer cases and deaths (128). Due to the fact that patients are often identified at a late stage, the recovery rate is poor. Despite ongoing advancements in colon cancer diagnosis and treatment methods, approximately 50% of patients still experience metastasis and recurrence (129, 130). Therefore, understanding the process of colon cancer cells is essential for creating novel tools for both diagnosis and treatment.

Transforming growth factor beta induced (TGFBI) takes involved in some of physiological processes, for example growth of the tumor, differentiation, and metastasis (131). The necessity of TGFBI in the development of cancer has been approved by numerous researchs. TGFBI is associated with a bad prognosis for patients and promotes the proliferation of oral squamous cell carcinoma cells. Overexpression of TGFBI promotes glioma cell proliferation and migration (132, 133). TGFBI may act as a pro- or anti-oncogenic molecule in ovarian cancer (134). Colon cancer tissues had higher levels of TGFBI mRNA, which is linked to extravasation and invasiveness (135, 136). For this reason, Gao and colleagues (53) analyzed how TGFBI affects colon cancer cells and whether miRNAs target the TGFBI gene. Using *in vitro* and bioinformatics experiments, it was determined that miR-766-3p can specifically aim TGFBI and that, in colon cancer, its level was downregulated while TGFBI was elevated. Additionally, they discovered that expression of miR-766-3p is forcefully suppressed cancerous cell activities and caused cell apoptosis in colon cancer. Overexpression of miR-766-3p inhibits cancer growth by targeting TGFBI (53). According to another research, it is intriguing to note that miR-766 may function as a microRNA that inhibits tumour growth in colon cancer cells. MiR-766 promotes the death of colon cancer cells *via* altering the p53/Bax signalling pathway (137). Consequently, miR-766 could be the new therapeutic aim for colon cancer.

Several tumor suppressor genes’ promoters are methylated, which directly inhibits transcription factor binding and halts gene expression (138). Elevated DNMT3B enzyme expression leads to aberrant de nevo methylation and CpG island methylation (CIMP) in colon cancer (139). MiR-339 and miR-766 target DNA Methyltransferase 3 Beta (DNMT3B) to alter methylation pattern in tumour suppressor genes (109). Ectopic production of miR-339 and specifically miR-766 has been shown to limit the growth of colon cancer by reducing the methylation that is in the tumor suppressor genes like Dickkopf-related protein 2 (DKK2), WNT inhibitory factor 1 (WIF1), secreted frizzled-related protein 1 (SFRP1), and SFRP2 (109).

#### 4.1.3 Breast cancer

Breast cancer is the most common type of cancer in women, leading to cancer-related death (128, 140). Individual particular targeted therapy has become more popular in therapeutic settings. Therefore, it is believed that identifying viable prognostic biomarkers and attractive targets is a necessary first step in achieving this goal. A tumor suppressor gene called P53 that frequently develops mutations in several different types of malignancies, for instance breast cancer. Changes in the gene impact the expression of several genes that are either directly or indirectly regulated by p53 (141) One of p53’s key roles is cell cycle regulation. Cell cycle arrest is predominantly caused by p53’s transactivation of its downstream targets, such as Stratifin (SFN), growth arrest and DNA damage-inducible alpha (GADD45A), and G2 and S phase-expressed-1 (GTSE1), at the G1/S and G2/M checkpoints (142). Previous research has shown that microRNAs have a function in p53-mediated control of the cell cycle. More and more microRNAs are being discovered as actors in the post-transcriptional regulation of p53 and the cell cycle regulation associated with p53 (143). Recently, Wang et al. (52), revealed that miR-766 expression levels were abnormally high in breast cancer and that miR-766 overexpression led to an increase of wild-type kind p53 protein in a number of cancer cell lines. In addition, miR-766 inhibits proliferation and engraftment in a variety of cancer cell lines and induces G2 cell cycle arrest, demonstrating its role in the p53 signalling pathway. Additionally, they discovered that the oncogene and negative regulator of p53 context-dependent roles of MDMX (MDM4) is specifically targeted by miR-766, which promotes p53 signaling (52). Overall, their research showed that miR-766 slows the spread of breast cancer through increasing p53 signaling by targeting MDM4.

Breast cancer that returns after therapy is referred to as a recurrence. Thirty-three percent of all patients with breast cancer will experience a distant metastatic relapse even after the initial disease remission. The primary factor in breast cancer patients’ deaths and a condition that is largely incurable is breast cancer metastasis (144, 145). Complex processes that makeup cancer metastasis is typically beyond the reach of a single molecular target’s intervention. A microRNA that regulates metastasis may be a promising option for a disease target since it may impact the aggressiveness of breast cancer cells by concurrently modulating many pathway effectors (146). By comparing a metastasis model with a patient-derived xenograft primary tumour model, Oh et al. (112) were able to evaluate the functional potential of a recently reported human metastasis-related miRNA, miR-766, which had been found before. In the orthotopic xenograft model, triple-negative breast cancer cells overexpressing miR-766 developed the primary tumour at the same pace as vector-transfected control cells. In contrast to control cancer cells, breast cancer cells that overexpressed miR-766 significantly inhibited the formation of tumour spheres and Matrigel invasion. In addition, compared to control cells, lung metastasis was significantly decreased in breast cancer cells overexpressing miR-766. They discovered that miR-766 upregulation dramatically lowers lung metastases in breast cancer (112). Therefore, miR-766 had a greater effect on distant metastasis than on cancer genesis and early tumour growth, and it may be a future therapeutic target for the effective treatment of lethal breast cancer metastasis.

#### 4.1.4 Renal cell carcinoma

Renal cell carcinoma (RCC), which accounts for 2% to 3% of adult malignancies, is the most lethal form of genitourinary cancer in humans (147). In around 30% of RCC patients, metastatic lesions are present at the time of first diagnosis (148–150). Deaths and morbidity from RCC have continued to grow in recent years. Surgical excision is still the only curative option for RCC because it is resistant to radiation, chemotherapy, and immunotherapy (151, 152). Although tyrosine kinase inhibitor (TKI)-based antiangiogenic therapy is the standard treatment for advanced or metastatic RCC, its efficacy is restricted owing to drug resistance (153, 154). Therefore, it is becoming more important to enhance novel RCC diagnostic and treatment procedures. Numerous miRNAs have been implicated in anti-tumor development in RCC patients, according to research done to date (155, 156). There is growing evidence that miR-766 either promotes or inhibits tumor growth in different group types of human malignancies. (**Table 1**). It’s intriguing to note that miR-766-3p is the miRNA that is downregulated the most in RCC, according to a genome-wide microRNA expression study (157). MiR-766-role 3p’s in RCC remains unknown. Alternate splicing regulators have acted as new participants in tumour regulating the activities of several tumour suppressors or oncogenes during the course of evolution (158, 159). The serine/arginine-rich (SR) proteins are crucial for alternative splicing regulation. Canonical component of the SR proteins, splicing factor SF2 is also known as SRSF1 (160, 161). If we observe SF2, it has bad prognosis and upregulation in a number of human malignancies (162, 163), It is still mostly unknown how SF2 and miRNAs interact. According to studies, RCC tissues had higher levels of methylation in the miR-766-3p promoter than non-tumorous tissues (116). In addition, it is evident that a decreased miR-766-3p level is associated with a poorer prognosis and clinical stage in RCC patients. A high level of SF2, a new oncoprotein in RCC, is highly associated with a poor prognosis in a large cohort of RCC specimens, and SF2 may stimulate the proliferation of RCC cells by boosting the expression of P-AKT and phosphorylated ERK (P-ERK). According to experimental findings, miR-766-3p may be specifically aiming SF2 in RCC cells (116). In addition, the expression of miR-766-3p ectopic inhibits cell division *via* targeting SF2 expression and other SF2/P-AKT/P-ERK signalling pathways. By modulating SF2/P-AKT/P-ERK signalling, miR-766-3p acted as an inhibitory noncoding RNA to restrict the progression of RCC (116). By focusing on this signaling pathway and miRNA, we may be able to better control the course of RCC and open a treatment path for additional RCC progression.

#### 4.1.5 Pancreatic cancer

Pancreatic cancer is one of the most lethal malignant tumours in the world. The most prevalent type of pancreatic cancer, is pancreatic ductal adenocarcinoma (PDAC), accounting for around 96% of all cases (164). The therapeutic results of PDAC patients remain poor despite the significant advancements achieved in therapeutic methods, such as surgery and radiochemotherapy (165). The lack of an effective early detection approach and the aggressive characteristics of PDAC, such as fast development and early metastasis, are mostly to blame for the poor prognosis of these individuals (166). PDAC patients generally have five-year survival rates of 5%, whereas those of patients who undergo surgical excision range from 10% to 25% (167, 168). Understanding PDAC processes is essential for developing novel biomarkers and therapeutic methods. According to accumulating evidence, several miRNAs are altered in PDAC, and these modifications have a significant effect on the malignant behavior of PDAC (169, 170). As a result, miRNAs are prospective therapeutic targets and diagnostic indicators for PDAC patients, and the relationship among miRNAs and PDAC has to be clarified. Recently, Li et al. (121) evaluated miR-766 expression in PDAC cell lines and tissues and looked into the mechanisms behind its functions in PDAC. They discovered that PDAC cell lines and tissues had considerably lower levels of miR-766. Additionally, PDAC cell invasion and proliferation were constrained by increased miR-766. Additionally, it was discovered that miR766 directly targeted E26 transformation specific 1 (ETS1) in PDAC cells. In addition, ETS1 overexpression negated the beneficial effects of miR-766 restoration on PDAC cells, while ETS1 downregulation mimicked the inhibitory impact of miR-766 overexpression on PDAC cells (121). MiR-766 has the potential to be a therapeutic target for PDAC patients. More studies are shown in **Table 1**.

### 4.2 miR-766 acts as oncomiRNA in cancer cells

According to several studies, miR-766 functioned as an oncomir that promoted cell migration and proliferation while inhibiting apoptosis by suppressing a number of targets. It was also found to be elevated in a number of malignancies (**Table 1**). MiR-766 was identified as an oncomir in the tissues of CRC patients with chronic myeloid, cutaneous squamous cell carcinoma (CSCC), leukemia, hepatocellular carcinoma (HCC), acute promyelocytic leukemia (APL), osteosarcoma, cervical cancer, breast cancer, and gastric cancer, as will be discussed further on.

#### 4.2.1 Cutaneous squamous cell carcinoma

Cutaneous squamous cell carcinoma is the second-most common kind of skin cancer. Despite tremendous advances in treatment approaches, the 5-year survival rate for cscc patients is insufficient due to the aggressiveness of the tumour and distant metastases (171). Consequently, it is essential to identify likely molecular pathways of CSCC development and to investigate potential therapeutic targets. PDCD5 is essential for cell proliferation, apoptosis, and metastasis (172, 173). On the other hand, miR-766 has been found to be significantly expressed in the CSCC patient’s cells and might be crucial to the disease’s molecular pathogenesis, according to a microarray analysis. On the CSCC, the precise regulatory impact of miR-766 is still unknown. Recently, Liu et al. (104), suggested that miR-766 might function in CSCC cells as an oncomiRNA. They discovered that in CSCC cells and tissues, miR-766 expression was elevated whereas PDCD5 expression was downregulated. MiR-766 sped up A431 and SCL-1 cells’ motility, invasion, and proliferation, while also boosting MMP-2 and MMP-9. As a result, miR-766 can increase proliferation migration and invasion, of CSCC cells by reducing the level of PDCD5 expression (104). Additionally, through increasing HOXA9, miR766-3p downregulation inhibited CSCC cell growth, metastasis, and glycolysis (174).

#### 4.2.2 Hepatocellular carcinoma

Hepatocellular carcinoma (HCC) is a leading cause of cancer mortality worldwide, with improved diagnosis and therapy. However, a substantial proportion of HCC patients are diagnosed at an advanced stage of the disease, and HCC recurrence or metastasis is still prevalent after liver resection (175). Therefore, a study on the abnormal expression and biological roles of miRNAs in HCC might lead to the creation of novel therapeutic targets and diagnostic biomarkers. Numerous research has examined the connections between miRNAs and the development of HCC illness in recent years (24, 176). However, only a few miRNAs were investigated in these studies, and it is necessary to evaluate the role of other miRNAs in the incidence of HCC. As was previously reported, miR-766 was linked to cell metastasis, proliferation, and death by targeting many down regulatory signaling pathways and genes in HCC cells (**Table 1**). However, more study is required to determine the molecular mechanisms and functions and of miR-766 in the development of HCC. Recently, Yang et al. (50) miR-766’s expression, functions, and processes in the *in vivo* and *in vitro* carcinogenesis of HCC were examined. They observed that miR-766 was higher in HCC tissues and that an increase in miR-766 directly reduces the expression of nuclear receptor subfamily 3 group c member 2 (NR3C2), hence promoting the spread and proliferation of HCC cells. In addition, they demonstrated that NR3C2 is one of the key targets of miR-766 and that it contributes to HCC carcinogenesis *via* altering the -catenin signalling pathway. This work established the importance of the miR766-NR3C2-catenin signalling pathway in the development of HCC and indicated that miR766 may play a role in the clinical treatment and diagnosis of HCC patients.

#### 4.2.3 Cervical cancer

Cervical cancer (CC) is a disease that affects the cervix and can lead to cancer (32, 96). The previous study predicted that CC annually results in about 509,590 new cases and 311,365 fatalities (128). Surgery and chemotherapy are effective for early-stage CC, but some patients experience metastasis and recurrence (177). An innovative method for treating CC and investigating its control mechanism (31, 178, 179).

A brand-new protein that is highly conserved, called SCAI (suppressor of cancer cell invasion) mediates metastasis by acting as a 3-dimensional matrix (180). According to research, SCAI serves as a target DNA for a number of miRNAs in human malignancies (181, 182). In many different types of cancer, SCAI is down-regulated and functions as an anti-tumor gene (183, 184). Cai et al. studied the miR-766-5p expression and its impact on CC initiation and progression (107). The research revealed that the expression levels of miR-766-5p and SCAI were significantly upregulated and downregulated, respectively, in CC tissues and cell lines. The receiver operating characteristic (ROC) curve suggests that miR-5p may be a biomarker for the diagnosis of CC, as it inhibits migration, proliferation, and invasion of CC cells while inducing apoptosis. By inhibiting SCAI, the inhibitory effects on the migration, invasion, and proliferation of CC cells *in vitro* were eliminated, and the inhibitory impact of SCAI on tumour xenograft growth *in vivo* was also eliminated (107).

## 5 Interactions among lncRNA/circRNA, with miR-766 in cancer

Noncoding RNAs regulate invasion, proliferation, apoptosis, and metastasis in cancer cells (185–187). Noncoding RNAs regulate the development of cancer in several ways. lncRNAs and miRNAs, for instance, alter target gene expression (mRNA), which is crucial for the biological activity of cancer cells (188). As opposing endogenous RNAs, lncRNAs and circRNAs have the binding affinity of miRNAs and interact with them, hence enhancing the translation of miRNA-targeted genes. MiRNAs, for example, inhibit the translation of their target genes by mRNA degradation or inhibition of mRNA translation. The biological basis of tumors is connected to the ceRNA regulatory network (189, 190). Additionally, circRNAs and lncRNAs can interact directly with proteins to influence gene transcription and so take part in regulation (191). The circRNA-lncRNA-miRNA-gene modification network in the apoptosis pathway of cancer has been the subject of several studies, which we sum up in this section. This research provides new insights into the pathogenic processes of cancer as well as novel views on the design of less invasive early detection and treatment methods.

### 5.1 CircularRNA and miR-766

CircRNAs are single-stranded, closed circular RNAs with poly (A) tails and 5′-3′ ends (192). Salzman et al. (193) More than 1/10 of genes that are have the potential to create circRNAs, according to research that quantified the quantity of circRNA types in both healthy and mammalian cells. Circular transcripts of CDR1 as act as miRNA sponges for miR-7 (194, 195). These studies made circRNAs the focus of scholarly investigation and the next big thing in the noncoding RNA area.

Nearly 184,615 new instances of laryngeal cancer were identified worldwide in 2018, and 99,840 people will pass away from the condition in 2020 (196). Laryngeal squamous cell carcinoma (LSCC), a prevalent kind of laryngeal cancer, imposes a substantial cost on individuals and the healthcare system (197). Similar to other cancers, the progression of LSCC is a complicated process including alterations to the metabolism, genes, and pathways (198, 199). However, the pathogenesis of LSCC hasn’t been precisely outlined. Consequently, it is essential to elucidate the molecular mechanism of LSCC. CircRNAs have crucial roles in the development of several malignancies, including LSCC (200) and circRNA-associated-ceRNA networks have discovered to be involved in LSCC development. CircRNA-associated-ceRNA networks have discovered to be involved in LSCC development (201, 202).

**Figure 3:**
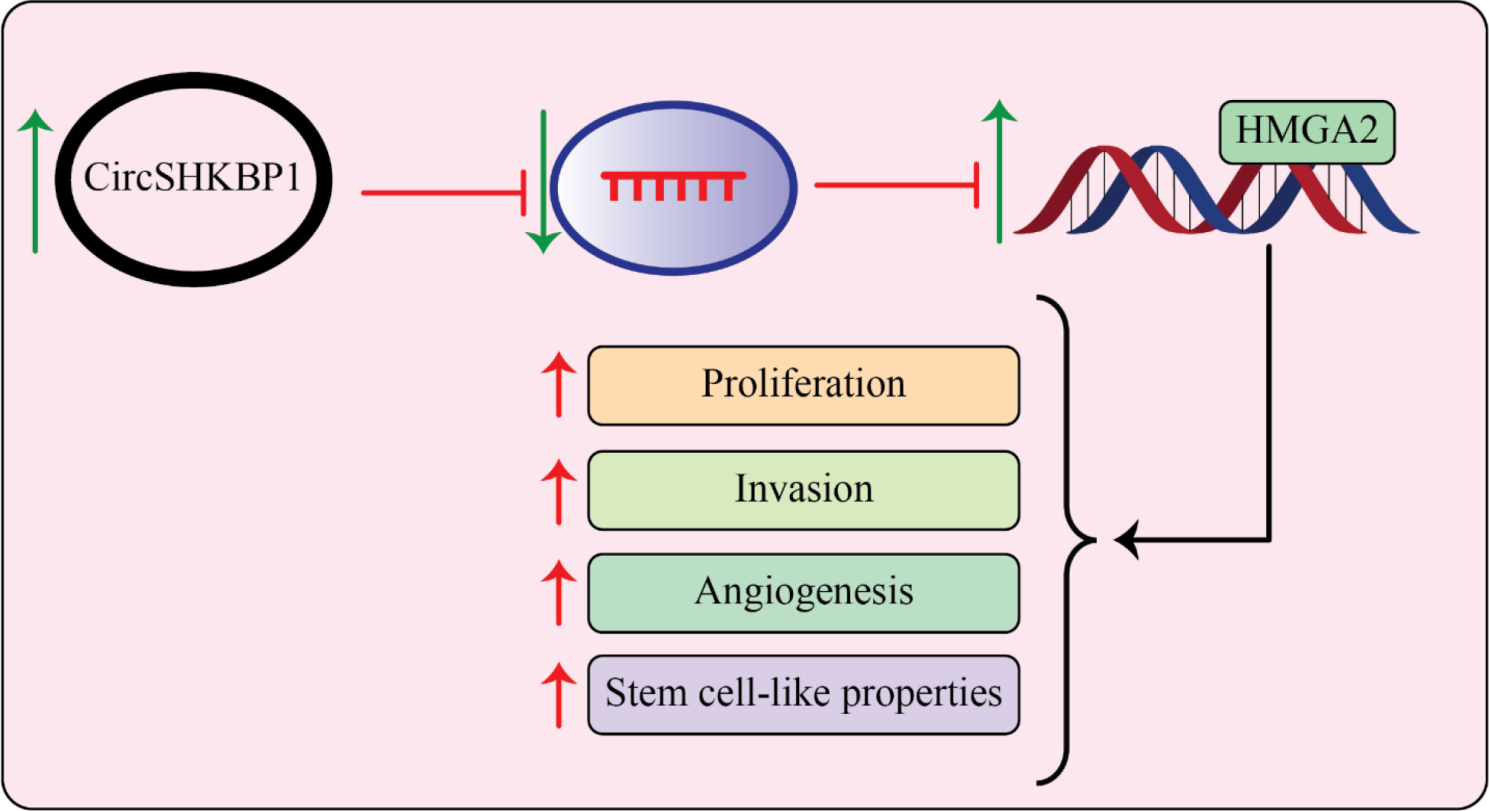
circSHKBP1 induces LSCC progression by upregulating HMGA2 levels by inhibiting miR-766-5p. The expression level of circSHKBP1 and miR-766-5p is upregulated and downregulated, respectively, in LSCC specimens and cell lines. High mobility group AT-hook 2 (HMGA2) promotes cancer development and miR-766-5p as tumor suppressor miRNA inhibits tumorigenesis of LSCC by targeting HMGA2. On another side, circSHKBP1 induces tumor development of LSCC by overexpression of HMGA2 through sponging miR-766-5p (120).

Recently, Chen et al. (120) LSCC progression was looked at to see if the circularRNA-associated ceRNA network involving circSHKBP1 and miR-766-5p was involved. They claimed that LSCC cell lines and specimens and showed up- and down-regulation of the genes circSHKBP1 and miR-766-5p, respectively. The expression of circSHKBP1 was associated with poor prognosis and clinical markers in LSCC, but decreased miR-766-5p expression improved the overall survival rate and could positively influence the high mobility group A2 (HMGA2). Importantly, circSHKBP1’s si-effects on the development of LSCC were abolished by HMGA2 overexpression. These findings revealed that circSHKBP1, which targets miR-766-5p, promoted LSCC carcinogenesis by increasing HMGA2 levels (**Figure 3**) (120).

Radiation therapy is a common and vigorous treatment for glioma, although its effectiveness is significantly hampered by the glioma cells’ innate and acquired resistance (203). Extracellular vesicles (EVs) contain circulating RNAs that are directly associated to the radioresistance of cancer cells (30, 74, 204, 205). In a study by Zhao et al. (206), to create radioresistant glioma U215 cells, glioma U251 cells were subjected to serial 5 Gy radiation treatments with a 60Co source (RR-U215). Following the separation of EVs as from RR-U125 cell culture media, an RT-qPCR study revealed that ciR-ATP8B4 expression was considerably higher in EVs than in non-EVs. The circular ATP8B4/miR-766-5p axis in RR-EVs may have a role in glioma radioresistance (206). Exosome-derived molecule makes HCT116 CRC cells more sensitive to chemotherapy drugs (207). MiR-766-5p is a sponge used for circ 0094343 and TRIM67 (tripartite motif-containing 67). It functions as an oncomiR in CRC tissues and has a negative correlation with TRIM67 expression. circ-0094343 enhances the chemosensitivity of CRC cells and inhibits their proliferation and glycolysis (207). Therefore, the circ 0094343/miR-766-5p/TRIM67 axis could provide novel insights into CRC treatment.

Cells’ stress response, known as autophagy, can shield them from harm (71). Incorrect autophagy has been seen in several diseases, including cancers and viral infections (71, 208). Moderate autophagy promotes invasion and development of pancreatic cancer cells, while excessive autophagy damages organelles and degrades pro-oncogenes, leading to apoptosis (209). Autophagy related 7 (ATG7) activation inhibits autophagy, reducing pancreatic cancer cell proliferation and metastasis (210, 211). Despite this, it is unknown how ATG7 governs pancreatic cancer.

Autophagy related 7 activation inhibits autophagy, reducing pancreatic cancer cell proliferation and metastasis (123). CircATG7 overexpression increased pancreatic cancer cell motility, proliferation, and autophagy, while ATG7 inhibition diminished its effects. MiR-766-5p works as a tumour suppressor to counter the effects of circATG7 (123). ATG7, a miR-766-5p target gene, was expressed greater, while circATG7 sponging miR-766-5p decreased its expression. As a conclusion, circATG7 speeds up the development of pancreatic cancer *via* miR-766-5p/ATG7 (123). circATG7 could therefore be a possible therapeutic approach for PC.

### 5.2 LncRNA and miR-766

Long non-coding RNAs play a significant role in the start, development, and metastasis of malignancies, and are emerging as potential biomarkers for cancer patients (70, 212). Additionally, the variety of cancers may have interesting therapeutic targets in lncRNAs and also the pathways which they affect. LncRNA nucleus enriched abundant transcript 1 (NEAT1), a crucial structural component of the subnuclear structure paraspeckle, has a close association with both innate immunity and malignant malignancies (213, 214). NEAT1 is involved in lymphoblastic, lymphoid, promyelocytic, and chronic myeloid leukemia (CML) cancers (213, 215, 216). However, its role in CML is not known. A large number of immature white blood cells accumulate in the bone marrow owing to CML, a malignant clonal proliferative disease that impairs the normal hematopoiesis of the bone marrow (217). Clinically, tyrosine kinase inhibitors (TKIs) are utilized to treat CML, despite their poor efficacy in patients with relapsed CML (218). Therefore, it is crucial to find new treatment targets for CML. In CML cell lines and PBMCs of CML patients, there is a considerable downregulation in the expression level of the lncRNA-NEAT1 (105). In addition, ectopic expression of NEAT1 reduces cell viability and enhances apoptosis in CML cells. In addition, NEAT1 overexpression decreased miR-766-5p expression in K562 and KCL22 cells, but NEAT1 knockdown increased it. MiR-766-5p levels are significantly elevated in CML patients. In addition, miR-766-5p reverses the effects of NEAT1 on CML cell growth and death, functioning as a tumour suppressor in these cells. Additionally, CDKN1A was identified as miR-766-target 5p’s gene and was inhibited in CML PBMCs. Overall, lncRNA-NEAT1 suppresses the growth of CML cells and stimulates apoptosis through sponging miR-766-5p. Upregulated miR-766-5p in response counteracted NEAT1’s impacts on apoptosis and survival rate in CML cells (105). A distinct finding was reported by Zhao et al. in a different study they conducted on the impact of the lncRNA-NEAT1/miRNA-766-5p axis in prostate cancer (PCa) cells (121). According to their findings, overexpressed miR-766-5p inhibits PCa cells’ ability to invade, migrate, and proliferate *via* aiming E2F transcription factor 3 (E2F3). In exchange, lncRNA-NEAT1 encourages the growth of PCa by sponging miR-766-5p (122). More research is therefore required on the NEAT1/miRNA-766-5p axis function in various malignancies.

Lung adenocarcinoma (LUAD), a subtype of lung cancer, has been recognised as a malignant lung tumour (147, 219). Numerous studies have shown that the development of LUAD includes a variety of complex biological processes, including numerous epigenetic and genetic alterations (220, 221). Despite substantial efforts in LUAD treatment, including chemotherapy, radiation, surgery, and molecular targeted therapy, the prognosis for individuals with severe LUAD remains bleak (222, 223). lncRNAs are associated with a number of biological processes in LUAD, including cell, invasion, metastasis, and proliferation, according to many investigations. LncRNA cancer susceptibility candidate 9.5 (CASC9.5), for example, stimulates the development and metastasis of LUAD cells (224). A new lncRNA called PRKCZ antisense RNA 1 (PRKCZ-AS1) has not been investigated in the growth of cancer. Wang et al. recently looked into the function of PRKCZ-AS1 in LUAD cells. In this work, it was observed that PRKCZ-AS1 expression was significantly upregulated in LUAD cell lines and tissues. Moreover, the inhibition of PRKCZ-AS1 reduced the migration and proliferation of LUAD cells while increasing death. Additionally, the expression of PRKCZ-AS1 in LUAD cells had a negative effect on the expression of miR-766-5p. In addition, overexpression of miR-766-5p in LUAD cells decreased cell migration and proliferation by targeting MAPK1 (119). In other words, PRKCZ-AS1 as onco-lncRNA promotes the development of LUAD by sponging miR-766-5p to boost MAPK1 expression, which improves the prospect of using PRKCZ-AS1 as a biomarker and therapeutic targets in LUAD research.

**Figure 4:**
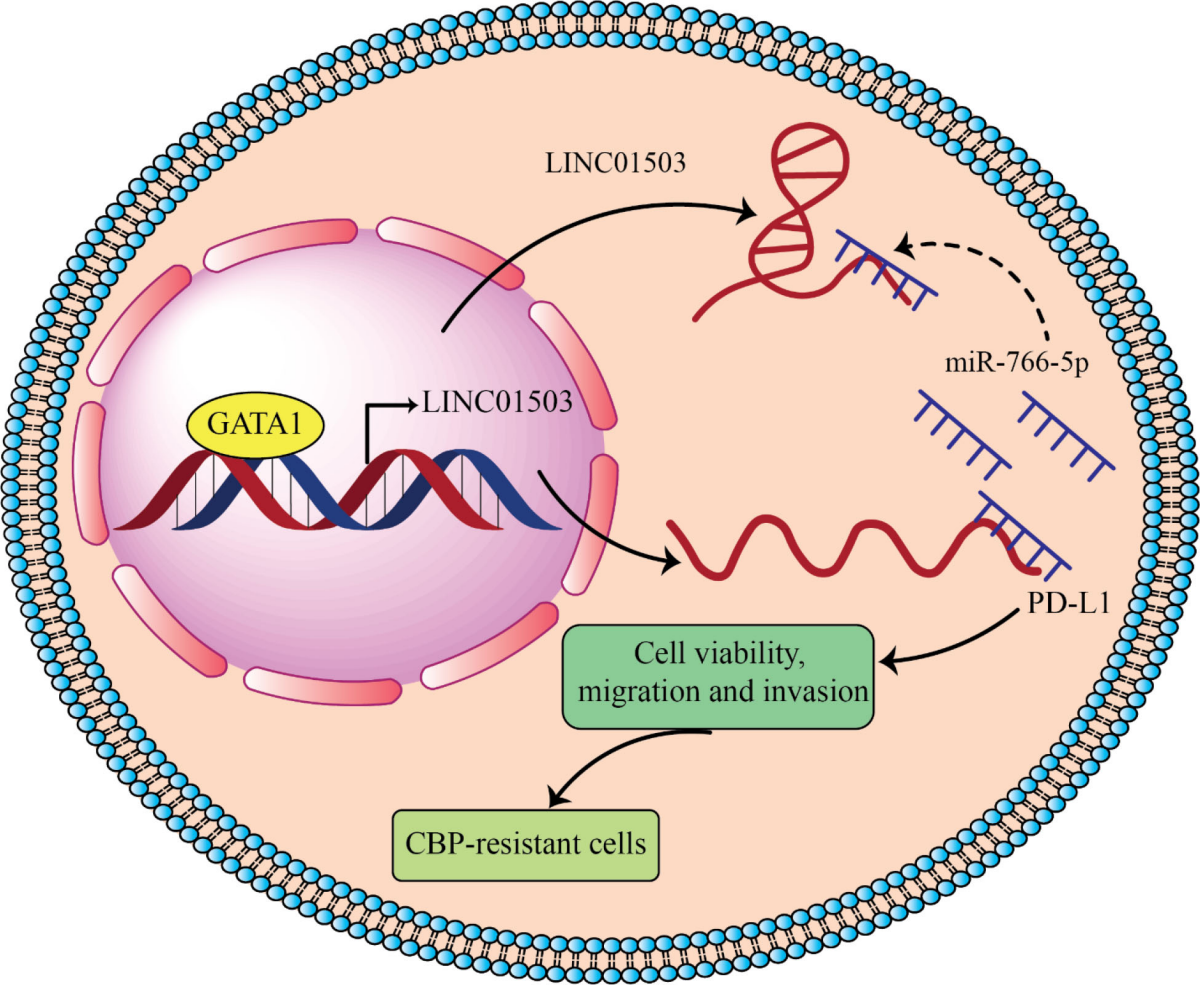
LINC01503 acts as onco-lncRNA in ovarian cancer and can induces cancer development by overexpressing PD-L1 through inhibiting miR-766 (87). LINC01503 and miR-766-5p expression levels significantly increase and decrease, respectively, in carboplatin (CBP) resistant ovarian carcinoma. Downregulation of LINC01503 decreases CBP resistance of ovarian carcinoma and in return overexpression of miR-766-5p increased CBP sensitivity by targeting PD-L1. GATA-binding protein 1 (GATA1) upregulated LINC01503 level and this lncRNA by sponging miR-766-5p leads to PD-L1 overexpression and promotes CBP resistance in OCa cells.

Recently, it has been observed that LINC01503 as an onco-lncRNA lead to inducing resistance of ovarian carcinoma to carboplatin by overexpressing PD-L1 through sponging miR-766-5p (87), which which can be used as potential target to overcome chemotherapeutic barriers in ovarian cancer hterapy (**Figure 4**).

## 6 MicroRNA Therapeutics in Cancer and limitation of miR-766

The miRNA replacement-based therapeutic strategy is directed at pathological cells that decrease the expression of onco-suppressor miRNAs that play a function in controlling the expression of mRNAs that encode crucial oncoproteins, using the development of anticancer treatments as a sample field of inquiry (225). It is obvious that important stage in oncogene upregulation leading to tumour initiation and advancement is the reduction of certain miRNAs that target oncogenes. MiRNA inhibitors and oligomers, such as small molecule inhibitors, miRNA sponges, miRNA masking, RNA, DNA, and DNA analogues (miRNA antisense treatment), can easily be used to suppress miRNA activity. On the other hand, miRNA mimetics that have been modified, like plasmid or lentiviral vectors expressing miRNA sequences, can be used to improve miRNA function (miRNA replacement treatment) (226).

For instance, Li et al. (207), who demonstrated that exosome-delivered circ 0094343 hinders the proliferation, clone formation, as well as glycolysis of colorectal cancer cells and improves chemosensitivity to different chemotherapeutic medications through the overexpression of TRIM67 through sponging miR-766-5p, described the inhibition of miRNA activity by miRNA sponges (207). In addition, it has been discovered that lenti-miR-766 transduction reduces cell proliferation and the population of colorectal cancer cell lines in the G1 phase *in vitro* (109). OS cells transfected with an inhibitor or mimic of miR-766-3p were injected subcutaneously into nude mice to examine miR-766-effects 3p’s *in vivo*. It was discovered that miR-766-3p knockdown increased OS cell development, resulting in larger and heavier tumors. Yet, high levels of miR-766-3p expression significantly slow tumor growth as compared to controls, resulting in a lighter and smaller tumor (227). In a different study, breast cancer cells that overexpress miR-766 (MB231 miR-766-GFP) formed less tumor spheres *in vivo* than control cells that had only been transfected with the vector (MB231 VectorGFP). In addition, compared to control cells, breast cancer cells with lung metastasis overexpressing miR-766 has been significantly decreased. Hence, miR-766 had a stronger impact on distant metastasis than Proliferation of cancer cells and early tumour growth, suggesting that tt might be a possible it could be a potential therapeutic target for the successful management of deadly breast cancer metastasis in the future (112).

On the basis of the observations that 1) the administration of various antagomiR molecules in combination causes stronger anti-tumor effects and 2) some anti-miR molecules can make drug-resistant tumour cell lines more susceptible to therapeutic drugs, combination strategies have recently been developed. A miRNA replacement treatment that combines antagomir and miRNA, and 3) miRNA replacement treatment combined with medication administration. The use of miR-766-based cancer therapy has not yet been studied, and more research is required.

There are certain restrictions on miR-766 that can be more carefully considered in further research. Although restoring normal miR-766 expression is advantageous, it is challenging to directly use miR-766 as a treatment due to the lack of information on the transcriptional and processing regulation of miR-766 during biogenesis and its precise function in tumorigenesis.

To reduce these adverse effects in an efficient treatment plan, specifics of the immunogenic and cytotoxic effects of miR-766 delivered *in vivo* must be investigated. Similar to how we suggested that miR-766 may be a part of the transcription factor-like gene regulatory network for mirnas, which is still unknown. As a result, it is not currently viable to knockdown inhibitory mirnas of miR-766 using anti-mirna oligos.

Limitations on Delivery Systems and Methods, a nother downside of a potential treatment is the lack of a suitable, secure, and efficient method of miR-766 distribution. Targeted delivery may employ biological vectors like aav and lentivirus, however uniformity of the procedure is necessary to avoid non-targeted site introduction. To treat brain and neuronal tumours, efficient neuron-specific delivery methods must be developed as delivery of mirna that targets the brain is not yet successful.

## 7 Conclusion

MiRNAs have received increasing attention as a result of their discovery, and one research discovered that they regulate a variety of cellular biological activities and contribute to the aetiology of disorders, including cancer (51). Additionally, the quantities of circulating miRNA are helpful for assessing or diagnosing human disease activities (51). This study investigated the role of miR-766 as a tumour suppressor or oncogene gene in relevant solid tumours, as well as its potential to be a tumour biomarker, targeted therapeutic method, and prognostic indicator in diverse cancer types. A number of studies have been conducted on the mechanisms underlying its activity as a cancer-inhibiting gene, but it is still unclear why it serves as both an onco-miRNA in cancer cells. This may be influenced by the pathogenic kind, histological origin, cellular microenvironment, and location of the relevant tumour.

In addition, we describe how lncRNAs and circRNAs act as sponges in cancer cells to regulate miR-766 and its target genes. We highlight examples of lncRNAs and circRNAs that interact with miR-766 in network modification and explore their impact on cancer malignancy. However, little is known about the mechanism by which the lncRNA-miR-766-mRNA ceRNA network controls the ncRNA field in cancer. Therefore, more research is required to clarify the activities and processes of cancer-related ceRNA. The circRNA, lncRNA, miRNA, and miRNA targeting mRNAs of the ceRNA network may potentially serve as potential therapeutic diagnostic markers and cancer targets. Future study will concentrate on clarifying previously reached controversial results.

## Author contributions

BF involved in the conception, design, and drafting of the manuscript. JG, HA-H, MA, AJ, MD, ZA, FA, AR-C and PR contributed to data collection and manuscript drafting. All authors approved the final version for submission.
